# Supplementary material for: The Impact of ZIP8 Disease-Associated Variants G38R, C113S, G204C, and S335T on Selenium and Cadmium Accumulations: The First Characterization
Source: Int J Mol Sci. 2021 Oct 22;22(21):11399. doi: 10.3390/ijms222111399 (PMC8583799; doi:10.3390/ijms222111399)
Supplement: Supplementary file 1 [file ijms-22-11399-s001.zip › ijms-1391568-Supplementary.pdf]

Supplementary Figures and Tables

The Impact of ZIP8 Disease-Associated Variants G38R, C113S, G204C, and S335T on Selenium and Cadmium Accumulations: The First Characterization

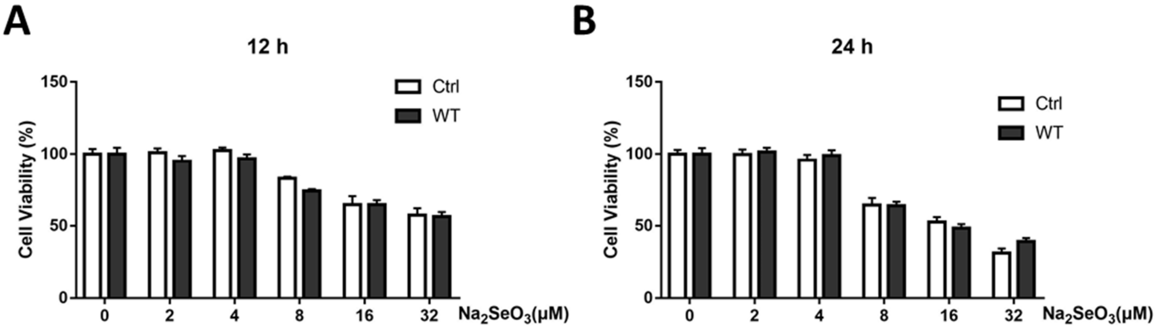

Figure S1. Effect of Se concentration on cell viability. (**A**,**B**) HeLa ZIP8-KO cells transient-transfected with pcDNA3.1 or pcDNA3.1-ZIP8-WT were treated with different doses of Na_2SeO_3 (0, 2, 4, 8, 16, and 32 μM) for (**A**) 12 or (**B**) 24 h, subsequently, NBB staining assay was carried out to detect the cell viability. The purity of Na_2SeO_3 (Sigma-Aldrich, Taufkirchen, Germany) used in this study is ≥98%.

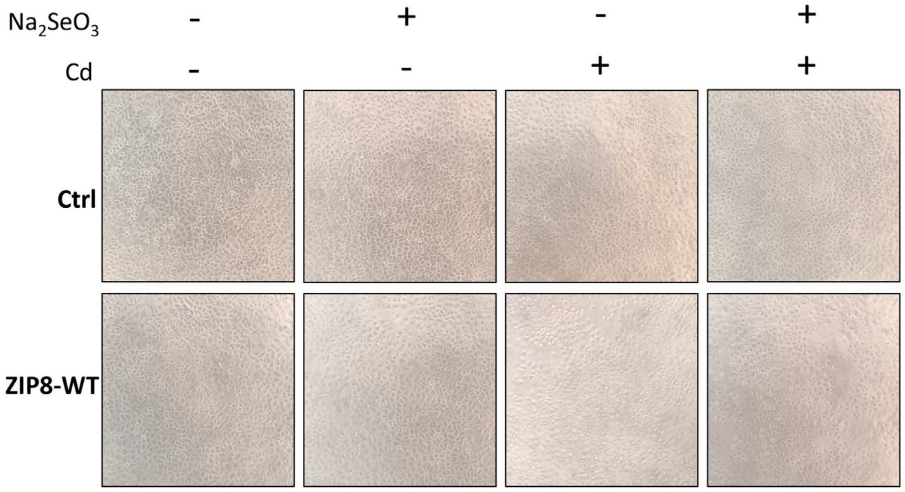

Figure S2. Morphology of HeLa ZIP8-KO cells overexpressing pcDNA3.1 or pcDNA3.1-ZIP8-WT after exposed to 4 μM of Na_2SeO_3 and/or 32 μM $CdCl_2$ for 12 h. The purity of $CdCl_2$ (Sigma-Aldrich, Taufkirchen, Germany) used in this study is 99.99%.

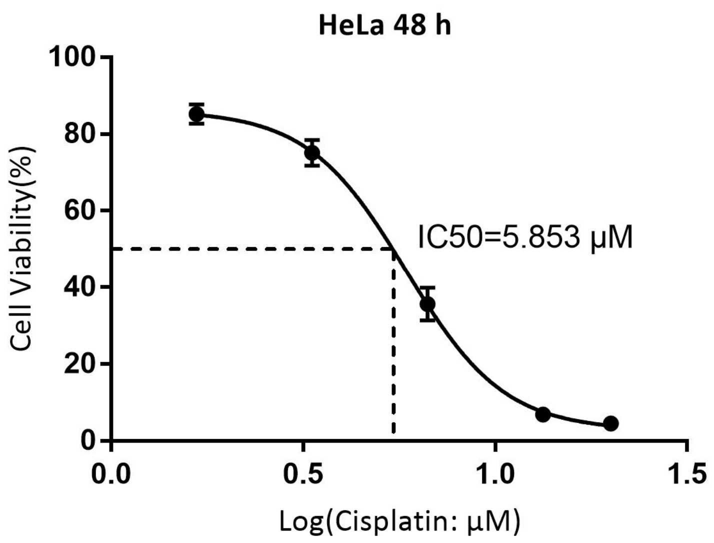

Figure S3. The dose-response curve of cisplatin in HeLa cells. HeLa cells were treated with different doses of cisplatin (0, 1.67, 3.33, 6.67, 13.33, and 20.00 μM) for 48 h, followed by NBB staining assay for detection of cell viability and IC50 value of cisplatin.

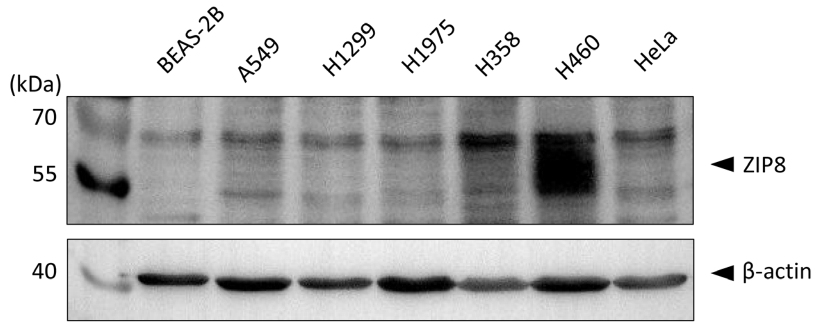

Figure S4. Protein expression of ZIP8 in normal lung epithelial BEAS-2B cells and cancer cell lines. Immunoblot analysis of ZIP8 in normal lung epithelial BEAS-2B cells and A549, H1299, H1975, H358, H460, and HeLa cancer cell lines. β-actin was used as the loading control.

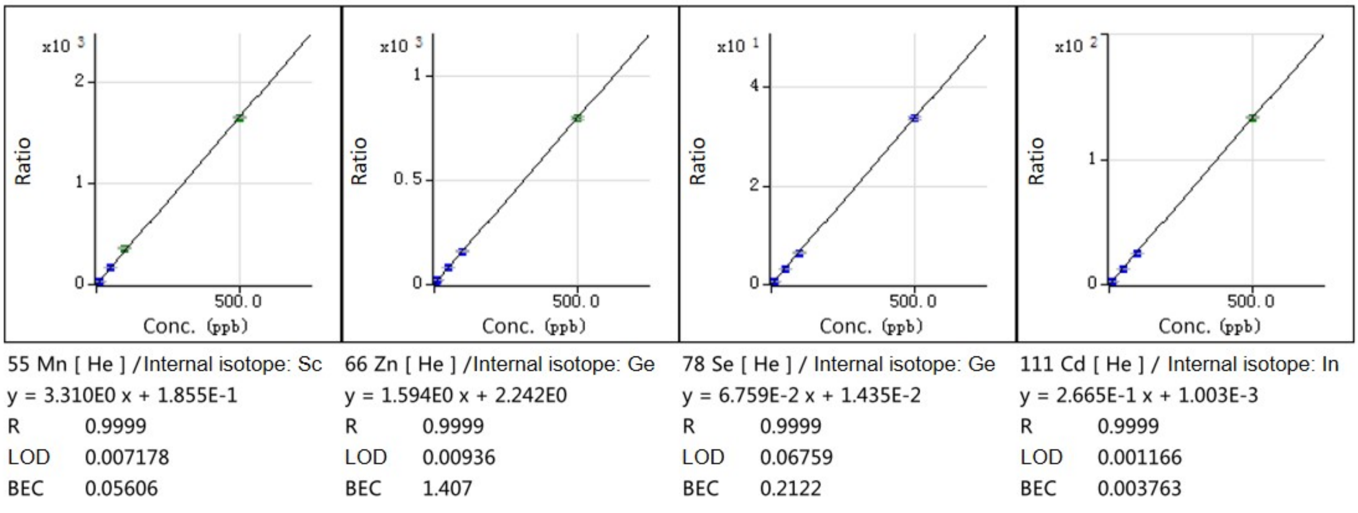

Figure S5. Detection results of the working standards curve of each detected element (Mn, Zn, Se, and Cd). The data include the internal isotopes, the limit of detection and correlation coefficients. BEC: Background Equivalent Concentration; LOD: Limit Of Detection.

Table S1. The PCR primers for verification of ZIP8-KO cells.

Primer	Sequence (From 5′ to 3′)
F	CCTTTAAACGCGCAATCCCC
R	AGTGTGCGAGCCGTTATCTT

Table S2. Cloning primer sets for sgRNA expression plasmids construction.

sgRNA(s)	Sequence (From 5′ to 3′)
sgRNA1-F	CACCGGCGTTTGCAAACCCGGATC
sgRNA1-R	AAACGATCCGGGTTTGCAAACGCC
sgRNA2-F	CACCGTGAACTGAGAGGTCCGACTC
sgRNA2-R	AAACGAGTCGGACCTCTCAGTTCAC

Table S3. The PCR primers for site-directed mutagenesis.

Mutation	Primer	Sequence (From 5′ to 3′)
ZIP8-G38R	F	CTGAGCGTGTTCCGCGCGAATCTGAGCCTGTCGG
ZIP8-G38R	R	GGCTCAGATTCGCGCGGAACACGCTCAGCACATCCTCG
ZIP8-C113S	F	AACTTTCACCCATCTGAGGATCGGCCCAAGCACAAAAC
ZIP8-C113S	R	GGGCCGATCCTCAGATGGGTGAAAGTTCAATTGCTGTA
ZIP8-G204C	F	AGTTGCTGTGTTTTGTGGATTTTACCTACTTTTCTTT
ZIP8-G204C	R	GTAGGTAAAATCCACAAAACACAGCAACTGCCTTCTCAA
ZIP8-S335T	F	CCTTCAGGGACTCACTACTTCCATAGCAATCCTATGTG
ZIP8-S335T	R	TTGCTATGGAAGTAGTGAGTCCCTGAAGGAGAGACAAGGT
